# Three-pedicle haemorrhoidectomy in the outpatient setting: the critical roles of information and organization

**DOI:** 10.3389/fsurg.2026.1748144

**Published:** 2026-02-13

**Authors:** Eleftherios Gialamas, Dior Marone, Amine Antonin Alam, Nadia Fathallah, Elise Pommaret, Vincent de Parades

**Affiliations:** 1 Institute of Proctology, Paris Saint-Joseph Hospital, Paris, France; 2 Division of Abdominal Surgery, Geneva University Hospitals, Geneva, Switzerland; 3 Faculty of Medicine, University of Geneva, Geneva, Switzerland

**Keywords:** ambulatory surgery, haemorrhoidectomy, organisational pathways, outpatient, patient satisfaction

## Abstract

**Introduction:**

Three-pedicle haemorrhoidectomy (Milligan-Morgan) has traditionally raised concerns in outpatient settings due to postoperative pain and complications. Since 2015, we have performed this procedure on an outpatient basis. This study aimed to assess patients' views on such care.

**Methods:**

We retrospectively included patients who underwent outpatient haemorrhoidectomy at our centre in 2020. A satisfaction questionnaire was sent. The primary outcome was the proportion of patients reporting good or excellent satisfaction. Secondary outcomes included hospital admissions, postoperative complications, and predictors of dissatisfaction. Ethics approval was obtained.

**Results:**

Among 392 patients, 292 underwent outpatient surgery (74%); 176 (60%) completed the questionnaire. Mean age was 52 ± 12 years; 64% were male. Good or excellent satisfaction was reported by 84% of respondents. Postoperatively, 9% required hospital admission, 48% contacted the hospital (nurse 52%, physician 48%) and 23% had unscheduled visits (clinic 65%, emergency department 35%). Complications occurred in 28% of cases, including discomfort, nausea, bleeding, urinary retention, and faecal impaction. Dissatisfaction was associated with poor preoperative explanations (surgeon *p* < 0.0001, anaesthetist *p* = 0.0005), complications (*p* = 0.0002), phone calls (*p* = 0.0016), and unscheduled visits (*p* = 0.0016). Multivariate analysis confirmed poor explanations by the surgeon (OR 0.08; *p* < 0.001) as an independent predictor. When asked, 79% said they would choose outpatient care again. Negative responses were independently associated with female sex (OR 0.33; *p* = 0.011), poor explanations (OR 0.11; *p* = 0.009) and unscheduled visits (OR 3.93; *p* = 0.02).

**Conclusions:**

Outpatient haemorrhoidectomy is acceptable to the majority of patients. However, thorough information and appropriate organisation are essential. As a result, 95% of these procedures are now performed on an outpatient basis at our centre.

## Introduction

Haemorrhoidal disease is one of the most common benign anorectal conditions, with a lifetime risk of up to 39% in the general population (1). Among the available surgical options, the Milligan & Morgan three-pedicle haemorrhoidectomy remains the gold standard for advanced (Goligher grade III–IV) or recurrent disease, providing durable symptom relief and low recurrence rates (2). However, this technique is also associated with significant postoperative morbidity, including pain, bleeding, urinary retention, and delayed functional recovery, factors that have traditionally limited its suitability for outpatient care (3).

Until the early 2010s, three-pedicle haemorrhoidectomy was performed exclusively as an inpatient procedure in most French institutions, including ours. Concerns over pain during the first bowel movement, bleeding risk, and urological complications largely dictated this cautious approach. A pivotal moment came in 2011 with the publication of national recommendations by the French Society of Digestive Surgery, which endorsed the feasibility and safety of performing excisional haemorrhoidectomy in an ambulatory setting (4). At the same time, the French Regional Health Agency (ARS) initiated a strong policy encouraging the development of ambulatory surgery across specialties.

In 2014, our team contributed to the development of national recommendations by the French Society of Coloproctology (SNFCP) supporting outpatient care for selected proctologic procedures, laying the foundation for institutional change (5). Consequently, in 2015 we began offering outpatient haemorrhoidectomy, supported by the implementation of a dedicated multimodal pain-management protocol. This included a preoperative nurse consultation, early scheduling to allow extended postoperative monitoring if needed, preoperative paracetamol, intraoperative pudendal nerve block with ropivacaine (6), routine intraoperative nonsteroidal anti-inflammatory drugs (NSAIDs), and systematic postoperative prescription of paracetamol, tramadol, and paracetamol–codeine. In parallel, we established a structured follow-up system to reassure patients and detect early complications (7, 8). A first prospective evaluation of this strategy, published in 2017, showed high satisfaction and acceptable complication rates in the initial cohort of 50 outpatient haemorrhoidectomy patients (9).

Building on these findings, this study aims to evaluate patient satisfaction with outpatient three-pedicle haemorrhoidectomy and to identify key factors, particularly those related to quality of information and organisational structure, that influence perception.

## Methods

### Study design and setting

This was a retrospective observational study conducted at the Proctology Department of Paris Saint-Joseph Hospital, a tertiary referral centre for medical and surgical proctology in France. The study was approved by the institutional ethics committee.

### Patient selection

All adult patients who underwent a conventional three-pedicle haemorrhoidectomy on an outpatient basis from 1 January to 31 December 2020 were screened for inclusion. Patients were identified through institutional coding databases (EGFA002 or EGFA003). Non-inclusion criteria were age <18 years, protected legal status (e.g., under guardianship), deprivation of liberty, refusal to participate, or inability to complete the satisfaction questionnaire.

### Surgical and anaesthetic protocol

All procedures were performed by senior proctologists, applying the standard three-pedicle haemorrhoidectomy, involving systematic excision of the three principal haemorrhoidal pedicles. No tailored, limited, or selective pedicle excision was performed in this cohort. A multimodal analgesia pathway was implemented, including preoperative paracetamol, intraoperative NSAIDs, and a pudendal nerve block using ropivacaine. General or spinal anaesthesia was chosen at the discretion of the anaesthetist during the pre-anaesthetic consultation. Patients received structured written and verbal preoperative information from the surgeon, anaesthetist, and dedicated preoperative nurse.

All patients were managed according to standard ambulatory surgery protocols, including post-anaesthesia monitoring in the recovery unit and the requirement for discharge with a responsible adult and home supervision on the day of surgery. In the recovery unit, pain was regularly assessed using a numerical rating scale (NRS) from 0 to 10 (0 = no pain, 10 = worst imaginable pain). For NRS values ≥3, morphine titration was initiated. Post-anaesthetic care unit discharge and subsequent hospital discharge were determined using the modified Aldrete score (10) and Chung score (11), respectively. Discharge criteria included stable vital signs, adequate pain control with oral analgesia, autonomous ambulation, and spontaneous urination. All patients were discharged on the same day and received a standardised oral and written postoperative care protocol.

### Postoperative care and follow-up

Postoperative analgesia at home followed a standardized multimodal regimen. All patients were prescribed scheduled paracetamol combined with non-steroidal anti-inflammatory drugs (NSAIDs), when not contra-indicated. Stepwise escalation to weak opioids (codeine or tramadol) was recommended when necessary. Patients were explicitly instructed to take analgesics systematically during the early postoperative period rather than on an as-needed basis, in order to anticipate pain escalation. Laxatives were routinely prescribed to prevent constipation-related complications.

Particular emphasis was placed on anticipating delayed postoperative pain and inflammatory symptoms, which were explained to patients during preoperative counselling as potentially peaking several days after surgery. Patients received clear instructions regarding expected symptom progression, warning signs, and appropriate use of analgesics. Patients received a systematic text message on postoperative day 1 to assess early pain and were provided with a contact number for both the nursing and surgical teams.

Follow-up consultation was routinely scheduled between postoperative days 10 and 20 in the outpatient clinic.

### Data collection

Patients were contacted by email or post between 6 and 12 months postoperatively and invited to complete a structured satisfaction questionnaire. The questionnaire included Likert-scale questions assessing satisfaction, quality of information received, postoperative complications, unplanned healthcare contacts, and willingness to undergo outpatient surgery again. Medical records were reviewed to extract demographic, clinical, and perioperative data.

### Outcomes

The primary outcome was the proportion of patients reporting good or excellent satisfaction with their outpatient experience. Secondary outcomes included postoperative complications (e.g., pain, bleeding, urinary retention, nausea, faecal impaction), hospital readmission, phone contacts and unscheduled consultations (in clinic or emergency department), predictors of dissatisfaction as well as willingness to undergo outpatient care again, and the trend to date in the proportion of three-quadrant haemorrhoidectomies performed as day-case procedures in the department.

### Statistical analysis

Categorical variables were reported as frequencies and percentages, while continuous variables were expressed as means ± standard deviation. Comparisons between groups (satisfied vs. dissatisfied) were performed using chi-square or Fisher's exact tests for categorical variables, and Student's *t*-test for continuous variables. Multivariate logistic regression models were constructed to identify independent predictors of dissatisfaction and refusal of future outpatient care. A *p*-value < 0.05 was considered statistically significant. Analyses were performed using R version 4.0.3 (R Foundation for Statistical Computing, Vienna, Austria).

## Results

During the 12-month study period, a total of 392 patients underwent three-pedicle haemorrhoidectomy, of whom 292 (74%) were operated on in the outpatient setting. Two patients refused to have their data processed and 176 patients (60%) completed the satisfaction questionnaire. The mean age was 52 ± 12 years, and 64% of respondents were male (Table 1).

**Table 1:** Univariate analysis of factors associated with patient satisfaction.

Variable	Total	Good/Excellent	Poor/Fair	OR	*p*-value
	(*N* = 176)	(*N* = 148, 84.1%)	(*N* = 28, 15.9%)	(95% CI)	
Age, years (mean ± SD)	51.9 ± 12.2	51.9 ± 12.1	52.4 ± 13.1	–	0.96
Sex					
Female	64 (36.4%)	52 (35.1%)	12 (42.9%)	1.00	0.44
Male	112 (63.6%)	96 (64.9%)	16 (57.1%)	0.72 (0.32–1.64)	
Diagnosis					
Prolapse	136 (77.3%)	115 (77.7%)	21 (75.0%)	1.00	0.85
Bleeding	31 (17.6%)	25 (16.9%)	6 (21.4%)	1.31 (0.48–3.59)	
Thrombosis	8 (4.6%)	7 (4.7%)	1 (3.6%)	0.78 (0.09–6.69)	
Other	1 (0.6%)	1 (0.7%)	0 (0.0%)	–	
Adequate explanation by surgeon					
No	12 (6.8%)	4 (2.7%)	8 (28.6%)	1.00	<0.0001
Yes	164 (93.2%)	144 (97.3%)	20 (71.4%)	0.07 (0.02–0.25)	
Adequate explanation by anaesthetist					
No	4 (2.3%)	0 (0.0%)	4 (14.3%)	1.00	0.0005
Yes	172 (97.7%)	148 (100.0%)	24 (85.7%)	–	
Complications (overall)					
No	126 (71.6%)	114 (77.0%)	12 (42.9%)	1.00	0.0002
Yes	50 (28.4%)	34 (23.0%)	16 (57.1%)	4.47 (1.93–10.36)	
Type of complication					
Bleeding	19 (10.8%)	13 (8.8%)	6 (21.4%)	2.83 (0.97–8.23)	0.09
Urinary retention	12 (6.8%)	10 (6.8%)	2 (7.1%)	1.06 (0.22–5.13)	>0.99
Nausea/vomiting	2 (1.1%)	2 (1.4%)	0 (0.0%)	–	>0.99
Faecal impaction	12 (6.8%)	6 (4.1%)	6 (21.4%)	6.45 (1.91–21.81)	0.0045
Stenosis	5 (2.8%)	4 (2.7%)	1 (3.6%)	1.33 (0.14–12.39)	0.58
Postoperative phone call					
No	92 (52.3%)	85 (57.4%)	7 (25.0%)	1.00	0.0016
Yes	84 (47.7%)	63 (42.6%)	21 (75.0%)	4.05 (1.62–10.11)	
Unscheduled consultation					
No	135 (76.7%)	120 (81.1%)	15 (53.6%)	1.00	0.0016
Yes	41 (23.3%)	28 (18.9%)	13 (46.4%)	3.71 (1.59–8.68)	
Readmission					
No	160 (90.9%)	139 (93.9%)	21 (75.0%)	1.00	0.0052
Yes	16 (9.1%)	9 (6.1%)	7 (25.0%)	5.15 (1.73–15.30)	

### Patient satisfaction

Patient satisfaction with outpatient care was high: 84% of respondents rated their experience as good or excellent, while 16% considered it mediocre, average, or poor (Table 1). Univariate analysis identified several factors significantly associated with dissatisfaction, including insufficient preoperative explanations provided by the surgeon and anesthetist, the occurrence of postoperative complications, particularly faecal impaction, phone contact with the care team, and unscheduled consultation (Table 1). In multivariate analysis, insufficient explanations provided by the surgeon was confirmed as independent predictor of dissatisfaction (Table 2).

**Table 2:** Multivariate analysis of predictors of dissatisfaction.

Variable	OR (95% CI)	*p*-value
Inadequate explanation by surgeon	0.08 (0.02–0.33)	<0.001
Complications	2.15 (0.74–6.27)	0.16
Postoperative phone call	2.13 (0.75–6.42)	0.16
Unscheduled postoperative consultation	1.91 (0.62–5.97)	0.26

Moreover, 79% stated that they would choose outpatient care again under similar circumstances, compared to 21% who expressed a preference for inpatient management in the future (Table 3). Univariate analysis identified several factors significantly associated with unwillingness to repeat the procedure on an outpatient basis, including female gender, insufficient preoperative explanations by the operator and anaesthetist, the occurrence of complications, phone contact with the care team, unscheduled consultation and hospital readmission (Table 3). Multivariate analysis confirmed that female gender, insufficient preoperative explanations by the surgeon and an emergency consultation were independent predictors of refusal to undergo repeat outpatient surgery (Supplementary Appendix 1).

**Table 3:** Univariate analysis of predictors of refusal to undergo outpatient haemorrhoidectomy again.

Variable	Would repeat	Would not repeat	OR	*p*-value
	(*N* = 138 78.9%)	(*N* = 37, 21.1%)	(95% CI)	
Age, years (mean ± SD)	52.0 ± 11.8	51.2 ± 13.5	–	0.72
Sex				
Female	44 (31.9%)	19 (51.4%)	1.00	0.029
Male	94 (68.1%)	18 (48.6%)	0.44 (0.21–0.93)	
Adequate explanation by surgeon				
No	4 (2.9%)	8 (21.6%)	1.00	0.0005
Yes	134 (97.1%)	29 (78.4%)	0.11 (0.03–0.38)	
Adequate explanation by anaesthetist				
No	1 (0.7%)	3 (8.1%)	1.00	0.030
Yes	137 (99.3%)	34 (91.9%)	0.08 (0.01–0.82)	
Complications				
No	104 (75.4%)	22 (59.5%)	1.00	0.06
Yes	34 (24.6%)	15 (40.5%)	2.09 (0.97–4.47)	
Postoperative phone call				
No	79 (57.3%)	13 (35.1%)	1.00	0.017
Yes	59 (42.8%)	24 (64.9%)	2.47 (1.16–5.26)	
Unscheduled postoperative consultation				
No	114 (82.6%)	20 (54.1%)	1.00	0.0003
Yes	24 (17.4%)	17 (45.9%)	4.04 (1.85–8.83)	
Readmission				
No	129 (93.5%)	30 (81.1%)	1.00	0.047
Yes	9 (6.5%)	7 (18.9%)	3.34 (1.15–9.70)	

### Complications

Postoperative complications were reported in 28% of cases. The most frequent issues included pain or discomfort, secondary bleeding, nausea or vomiting, urinary retention, and faecal impaction (Supplementary Appendix 2). No mortality or long-term sequelae were observed during follow-up. Nearly half of the respondents (48%) contacted the hospital following surgery, either via the nursing coordination team (52%) or through surgical residents (48%). A total of 23% of patients returned for an unscheduled consultation and 9% of patients required unplanned hospital readmission.

The majority of complications were managed conservatively, either through analgesic adjustment, or outpatient consultation. Only a limited proportion required unplanned consultation or hospital readmission, and no life-threatening complications, mortality, or long-term sequelae were observed.

### Institutional trend

**Figure 1:**
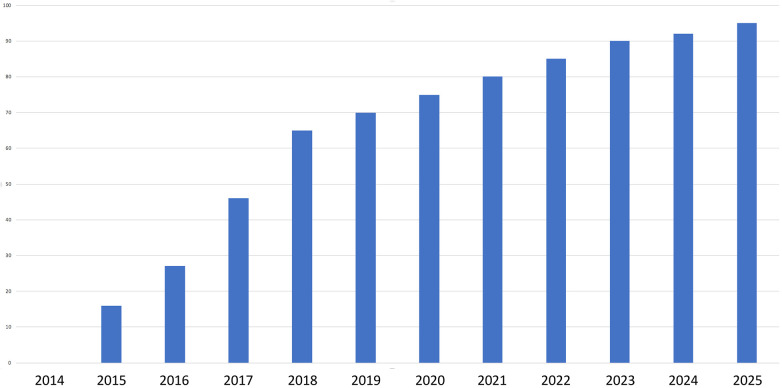
Evolution of outpatient three-pedicle haemorrhoidectomy at Paris Saint-Joseph hospital between 2014 and 2025.

Institutional data showed an increase in the proportion of haemorrhoidectomies performed on an outpatient basis. From 0% in 2014, this rate rose progressively to 95% by 2025. This evolution is illustrated in Figure 1.

## Discussion

Our study demonstrates that outpatient three-pedicle haemorrhoidectomy is both feasible and well tolerated by the vast majority of patients when integrated into a structured, multidisciplinary perioperative care pathway. We observed this among a typical population of patients with haemorrhoidal disease, which is particularly interesting given that one might assume that satisfied patients would not have seen fit to respond to the questionnaire we sent them. Furthermore, institutional data showed an increase in the proportion of haemorrhoidectomies performed on an outpatient basis. From 0% in 2014, this figure rose progressively to 95% by 2025, reflecting the structured implementation of multimodal pain management and organisational optimization in our department for these patients. This is particularly noteworthy given the long-standing perception of the Milligan-Morgan three-pedicle haemorrhoidectomy as too painful or risky for ambulatory settings. Previous literature has documented concerns related to postoperative pain, urinary retention, and secondary bleeding as barriers to same-day discharge. Our findings support more recent evidence that, when carefully implemented, outpatient excisional haemorrhoidectomy can be performed without increased morbidity or a decline in patient satisfaction (9, 12–14).

Pain management remains a cornerstone of this transformation. Our institutional multimodal analgesic protocol, combining preoperative education, pre-emptive paracetamol, intraoperative pudendal nerve block with ropivacaine, intra- and perioperative NSAIDs, and postoperative prescriptions, enabled same-day discharge with acceptable pain control and low readmission rates. These findings align with high-level evidence showing that pudendal nerve blocks significantly reduce early postoperative pain and opioid consumption, while perioperative NSAIDs contribute to overall analgesic effectiveness (6, 15–17). Importantly, this approach aligns with enhanced recovery after surgery (ERAS) principles, which emphasise multimodal analgesia and early mobilisation (18).

A distinctive element of our study lies in the detailed analysis of patient satisfaction determinants. Multivariate modelling revealed that insufficient preoperative explanations, whether by the surgeon or the anaesthetist, were independent predictors of dissatisfaction. These findings mirror prospective studies in colorectal and general surgery, which consistently report that clear expectation-setting, comprehensive perioperative counselling, and accessible postoperative contact points improve satisfaction and reduce unplanned consultations (7, 19–21). Interestingly, while complications occurred in nearly one-third of our cohort, they were not the strongest predictor of dissatisfaction. This suggests that patients are more willing to accept complications when they feel adequately informed and supported.

A substantial proportion of patients contacted healthcare providers or returned for unscheduled consultations after discharge. These events likely reflect a combination of expected reassurance-seeking behaviour and symptom-related concerns rather than severe postoperative morbidity, particularly within the context of the French healthcare system. Within an outpatient care model, such contacts were anticipated and facilitated through structured accessibility to nursing and surgical teams, which may represent a strength of the pathway rather than a failure of outpatient management.

Among postoperative complications, faecal impaction was particularly distressing and often triggered emergency consultations. This observation is in line with smaller series and ambulatory proctology audits reporting that constipation-related events, although rarely serious, are disproportionately impactful on patient experience (22–24). Structured postoperative dietary guidance, early laxative prescription, and proactive follow-up may mitigate these events.

Another notable finding was the influence of sex on patient perceptions. Female patients were significantly more likely to report that they would not choose outpatient care again. While the reasons for this are multifactorial, hypotheses include differing pain perception, greater preoperative anxiety, or different postoperative social support structures. The literature on gender differences in surgical satisfaction is mixed, with some studies showing no association and others suggesting that women report lower satisfaction in high-discomfort procedures (25, 26). This highlights the need for tailored preoperative counselling that addresses patient-specific concerns.

From an institutional standpoint, our experience illustrates the role of organisation and culture in driving sustainable change. Since the introduction of our dedicated outpatient pathway in 2015, supported by clear analgesic protocols, coordinated scheduling, and structured follow-up, outpatient rates have risen steadily to reach 95% in 2025. This transition was gradual, relying on iterative protocol refinements, interdisciplinary engagement, and continuous monitoring of outcomes. Crucially, this shift did not result in increased postoperative morbidity or a decline in satisfaction, confirming that cultural change can be achieved without compromising safety. Our findings have broader implications for other centres aiming to expand their ambulatory proctology services. They underline that success is not solely dependent on surgical technique but requires investment in preoperative patient education, multidisciplinary coordination, and robust postoperative support systems. These elements are increasingly recognised in surgical quality frameworks as being as important as intraoperative performance.

Our study has some limitations. Its retrospective design limits causal inference and is susceptible to information bias. Additionally, this is a single-centre study conducted in the largest and most active proctology unit in France, which may limit external generalisability. This work was also carried out during the first wave of COVID-19; in this particular context, it did not modify our hospitalisation procedures, and it is difficult to say whether it had any impact on patients' feelings about having their surgery performed on an outpatient basis. Furthermore, postoperative complications were not classified using a standardized severity grading system (such as Clavien–Dindo), which limits the precise assessment of their clinical impact and comparability with other series. On the other hand, the size of the cohort, the maturity of the outpatient pathway, and the reproducibility of the organisational model strengthen the relevance of our findings for similar high-volume centres. Finally, although economic outcomes represent an important dimension of ambulatory surgery, the present study was not designed to assess costs or resource utilisation, which were therefore not analysed.

## Data Availability

The raw data supporting the conclusions of this article will be made available by the authors, without undue reservation.
