# Supplementary material for: Three-pedicle haemorrhoidectomy in the outpatient setting: the critical roles of information and organization
Source: Front Surg. 2026 Feb 13;13:1748144. doi: 10.3389/fsurg.2026.1748144 (PMC12946137; doi:10.3389/fsurg.2026.1748144)
Supplement: Supplementary file 1 [file Table1.docx]

Supplementary Material

**Appendix 1: Multivariate analysis of predictors of refusal to undergo outpatient haemorrhoidectomy again**

| Variable | OR [95% CI] | p-value |
| --- | --- | --- |
| Female sex | 0.33 [0.13; 0.77] | 0.0114 |
| Inadequate explanation by surgeon | 0.11 [0.02; 0.56] | 0.0087 |
| Inadequate explanation by anaesthetist | 0.39 [0.01; 6.50] | 0.52 |
| Complications | 0.58 [0.19; 1.65] | 0.32 |
| Postoperative phone call | 1.26 [0.48; 3.29] | 0.63 |
| Unscheduled postoperative consultation | 3.93 [1.32;12.26] | 0.0153 |
| Readmission | 3.69 [0.92;14.80] | 0.06 |

**Appendix 2: Comparison of patients with and without complications**

| Variable | No complications  (N = 126, 71.6%) | Complications  (N = 50, 28.4%) | OR  [95% CI] | p |
| --- | --- | --- | --- | --- |
| Age, years (mean ± SD) | 51.7 ± 12.2 | 52.6 ± 12.2 | – | 0.65 |
| Sex |  |  |  |  |
| Female | 46 (36.5%) | 18 (36.0%) | 1.00 | 0.95 |
| Male | 80 (63.5%) | 32 (64.0%) | 1.02 [0.52–2.02] |  |
| Adequate explanation by surgeon |  |  |  |  |
| No | 5 (4.0%) | 7 (14.0%) | 1.00 | 0.040 |
| Yes | 121 (96.0%) | 43 (86.0%) | 0.25 [0.08–0.84] |  |
| Adequate explanation by anaesthetist |  |  |  |  |
| No | 1 (0.8%) | 3 (6.0%) | 1.00 | 0.07 |
| Yes | 125 (99.2%) | 47 (94.0%) | 0.13 [0.01–1.24] |  |
| Type of complication |  |  |  |  |
| Bleeding | 0 (0.0%) | 19 (38.0%) | – | <0.0001 |
| Urinary retention | 0 (0.0%) | 12 (24.0%) | – | <0.0001 |
| Nausea/vomiting | 0 (0.0%) | 2 (4.0%) | – | 0.08 |
| Faecal impaction | 0 (0.0%) | 12 (24.0%) | – | <0.0001 |
| Stenosis | 0 (0.0%) | 5 (10.0%) | – | 0.0016 |
| Postoperative phone call |  |  |  |  |
| No | 79 (62.7%) | 13 (26.0%) | 1.00 | <0.0001 |
| Yes | 47 (37.3%) | 37 (74.0%) | 4.78 [2.31–9.91] |  |
| Unscheduled postoperative consultation |  |  |  |  |
| No | 114 (90.5%) | 21 (42.0%) | 1.00 | <0.0001 |
| Yes | 12 (9.5%) | 29 (58.0%) | 13.12 [5.79–29.73] |  |
| Readmission |  |  |  |  |
| No | 122 (96.8%) | 38 (76.0%) | 1.00 | <0.0001 |
| Yes | 4 (3.2%) | 12 (24.0%) | 9.63 [2.93–31.62] |  |
